# Fraser Syndrome in a Newborn: Diagnostic Challenges Based on Clinical Criteria

**DOI:** 10.7759/cureus.107268

**Published:** 2026-04-17

**Authors:** Kaoutar Ettoini, Khadija Mesbah, Yousra Elboussaadni, Abdallah Oulmaati

**Affiliations:** 1 Pediatrics, Centre Hospitalier Universitaire Mohammed VI – Tanger, Tangier, MAR

**Keywords:** anophthalmia, clinical diagnosis, fras1 gene, fraser syndrome, genital anomalies, polymalformative syndrome, rare genetic disorder, syndactyly

## Abstract

Fraser syndrome is a rare autosomal recessive congenital malformation syndrome characterized by a wide clinical spectrum, including ocular, limb, genital, and visceral anomalies. Diagnosis is suggested by the association of multiple characteristic malformations.

We report the case of a newborn admitted to the Neonatology and Neonatal Intensive Care Unit of Mohammed VI University Hospital in Tangier, born to consanguineous parents, who presented with a polymalformative syndrome associating bilateral anophthalmia with ankyloblepharon, syndactyly, genital anomaly, anal imperforation, and low-set ears. Additional ultrasound assessment revealed associated cardiac and genital abnormalities, without major renal involvement. The overall clinical and radiological findings were highly suggestive of Fraser syndrome.

This observation highlights the phenotypic heterogeneity of Fraser syndrome and emphasizes the diagnostic value of careful clinical evaluation in the presence of multiple congenital malformations, particularly in a context of consanguinity. Early prenatal suspicion may help optimize perinatal management and parental counseling. Management is multidisciplinary and involves neonatologists, pediatric surgeons, geneticists, and long-term follow-up. Prognosis depends on the type and severity of associated malformations and remains guarded in severe presentations.

## Introduction

Fraser syndrome is a rare autosomal recessive congenital disorder characterized by a wide spectrum of polymalformative anomalies. First described by George Fraser in 1962 [1], its estimated prevalence is approximately 0.43 per 100,000 live births [2]. The syndrome is classically defined by the association of cryptophthalmos (a congenital anomaly in which the eyelids fail to separate), syndactyly (fusion of digits), and urogenital malformations, often accompanied by laryngeal, renal, and craniofacial anomalies [3-4].

From a genetic perspective, Fraser syndrome is genetically heterogeneous and is mainly associated with mutations in the *FRAS1*, *FREM2*, and *GRIP1 *genes [5]. These genes encode proteins involved in epithelial-mesenchymal adhesion during embryonic development. Disruption of this adhesion process leads to defective morphogenesis, particularly affecting structures that require epithelial separation, thereby explaining the characteristic anomalies such as cryptophthalmos and syndactyly [6].

Clinically, the diagnosis of Fraser syndrome is primarily based on established clinical criteria, notably those proposed by Thomas et al. [7], which combine major and minor anomalies. However, the diagnosis remains challenging due to significant phenotypic variability and overlap with other congenital malformation syndromes. In contemporary clinical genetics, molecular confirmation is considered the gold standard; nevertheless, in many settings, particularly in resource-limited countries, access to genetic testing may be restricted, making clinical diagnosis still highly relevant.

Fraser syndrome is associated with significant morbidity and mortality, especially in cases involving renal agenesis or laryngeal atresia, which can be life-threatening in the neonatal period. Early recognition is therefore essential for appropriate management, multidisciplinary care, and genetic counseling.

We report a case of a newborn presenting with a severe polymalformative phenotype highly suggestive of Fraser syndrome. The originality of this case lies in the completeness of the clinical presentation and the diagnostic approach based on established clinical criteria in a resource-limited setting, highlighting both the challenges and the relevance of clinical assessment in the absence of molecular confirmation.

## Case presentation

Anamnesis

This report concerns a newborn of indeterminate sex, admitted on the first day of life to the neonatal intensive care unit for evaluation of a polymalformative syndrome.

The patient was born to consanguineous parents (first degree). The maternal history revealed gravida 9 para 3 (G9P3), with two living healthy children, three stillbirths, and three miscarriages of unclear etiology. The pregnancy was poorly monitored and carried to 35 weeks of gestation, with no antenatal anomaly scan performed. Maternal serologies (HIV, rubella, hepatitis B and C) and toxoplasmosis screening were negative. No history of drug exposure, radiation, or toxic substances during pregnancy was reported.

In our patient, the association of three major criteria (bilateral anophthalmia, syndactyly, and genital anomalies) and two minor criteria (low-set ears and imperforate anus) strongly supported a clinical diagnosis of Fraser syndrome according to established diagnostic criteria [7]. Delivery was vaginal, with good adaptation to extrauterine life.

Methods

Clinical Examination

**Figure 1:**
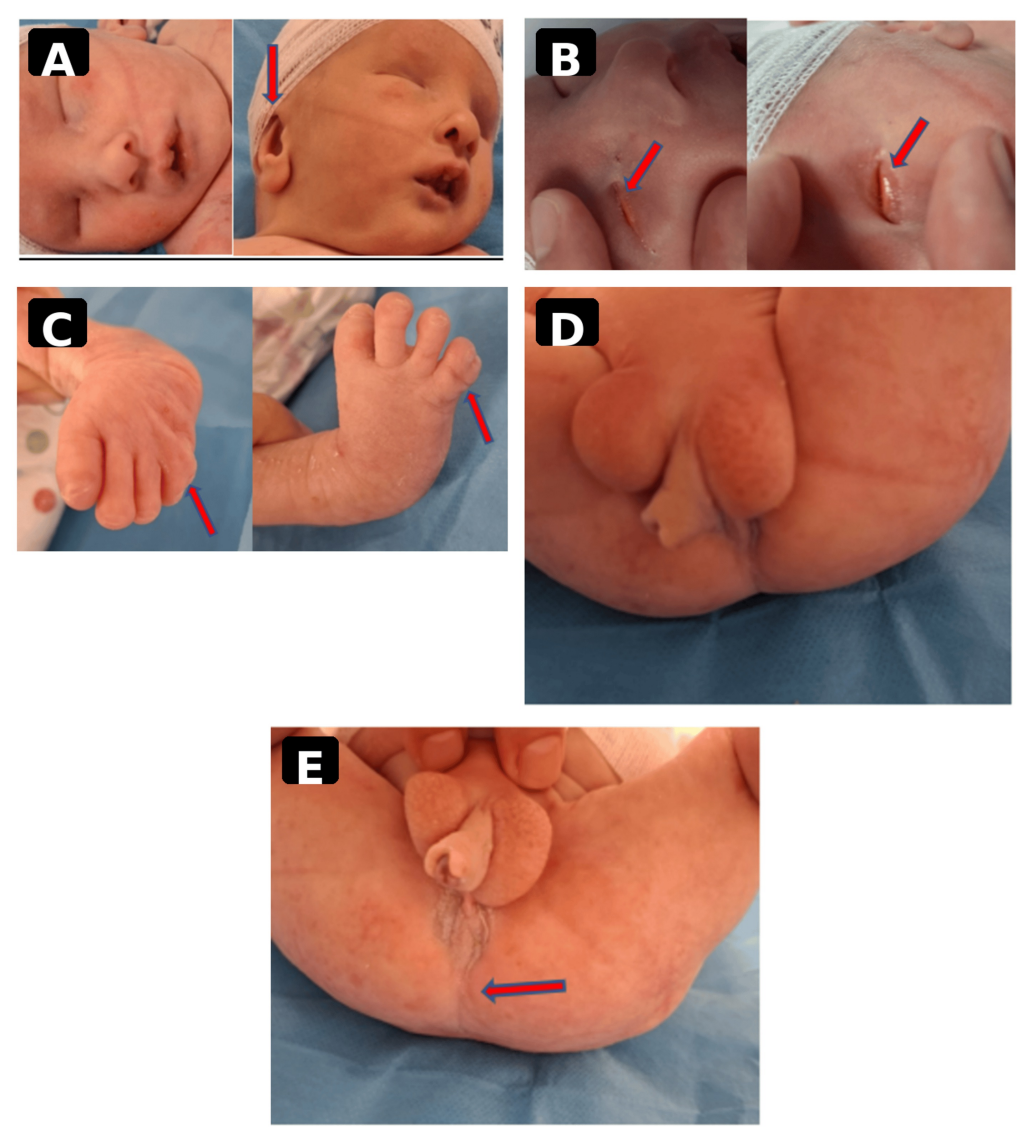
Composite clinical image showing characteristic features of Fraser syndrome in the reported newborn. (A) Craniofacial view showing microcephaly, short neck, and low-set ears (arrows). (B) Bilateral anophthalmia with ankyloblepharon, characterized by the absence of palpebral fissure opening and absence of identifiable ocular structures (arrows). (C) Bilateral syndactyly of the 4th and 5th toes, illustrating cutaneous fusion of the interdigital spaces (arrows). (D) External genital anomalies, including a scrotal-like appearance, clitoromegaly, and labial hypoplasia (arrows). (E) Imperforate anus, observed on perineal examination (arrow).

On admission, the newborn was hemodynamically stable, with normal respiratory parameters (Silverman score 0/10, oxygen saturation of 96% in room air). Anthropometric measurements were as follows: birth weight - 1800 g (10th percentile), length - 42 cm (3rd percentile), and head circumference - 32 cm (50th percentile). These measurements, interpreted according to gestational age-appropriate growth charts [8], indicated asymmetric intrauterine growth restriction. Physical examination revealed polymalformative syndrome, including microcephaly, short neck, and low-set ears (Figure 1A), bilateral anophthalmia with ankyloblepharon (Figure 1B), syndactyly involving the 4th and 5th toes bilaterally (Figure 1C), genital anomalies (scrotal-like appearance, clitoromegaly, incomplete labial development) (Figure 1D), and imperforate anus (Figure 1E).

Complementary investigations

Complementary investigations revealed the following: abdominopelvic ultrasound confirmed the presence of both kidneys, with the uterus visualized but not communicating with the vaginal cavity. Ocular ultrasound showed the absence of identifiable ocular structures. Cardiac ultrasound demonstrated an intermediate atrioventricular septal defect with moderate pulmonary hypertension and a patent ductus arteriosus measuring 4 mm. Transfontanellar ultrasound was normal, and thoracoabdominal X-ray was unremarkable. Laboratory findings included a normal electrolyte panel and normal 17-hydroxyprogesterone. Karyotype was 47,XX.

Differential diagnosis

Given the polymalformative presentation, several differential diagnoses were considered: isolated forms of cryptophthalmos, Lenz microphthalmia syndrome, oculo-dento-digital dysplasia, short rib-polydactyly syndromes, and other ciliopathies, and complex multiple congenital anomaly syndromes involving urogenital and anorectal malformations. However, the characteristic association of major ocular anomalies (Figure 1B), syndactyly (Figure 1C), and genital anomalies (Figure 1D) strongly supported the diagnosis of Fraser syndrome based on established clinical criteria [7].

Results

The patient fulfilled three major criteria (ocular anomaly within the cryptophthalmos spectrum, syndactyly, and genital anomaly) and two minor criteria (low-set ears and imperforate anus), as illustrated in Figure 1A-1E, supporting a clinically highly suggestive diagnosis of Fraser syndrome [7]. Initial management consisted of clinical stabilization and the organization of multidisciplinary care.

Outcome and Follow-Up

A multidisciplinary management approach was initiated, involving neonatology, pediatric surgery, cardiology, and genetics teams. Surgical interventions were planned for syndactyly (Figure 1C), genital anomalies (Figure 1D), and anorectal malformation (Figure 1E). Genetic counseling was recommended for the family due to consanguinity and the associated risk of recurrence.

The short-term clinical course was stable. Long-term follow-up is required to assess growth, neurodevelopmental outcomes, and complications related to associated malformations.

## Discussion

Fraser syndrome is a rare autosomal recessive congenital disorder characterized by a wide spectrum of malformations involving ocular, limb, genital, and visceral structures, with more than 350 cases reported in the literature [2,7,9]. Its pathogenesis remains incompletely understood, although the most widely accepted hypothesis involves a disruption of programmed cell death during embryogenesis, leading to persistence of epithelial adhesions and failure of normal anatomical separation. Alternative mechanisms, including primary abnormalities in epithelial development, have also been proposed [2].

Consanguinity, reported in approximately 15-25% of cases, supports the autosomal recessive inheritance pattern and contributes to increased disease occurrence in certain populations [5,9]. This was also observed in our case, emphasizing the importance of genetic counseling, particularly in regions where consanguineous marriages are more prevalent.

Fraser syndrome is caused by mutations in the *FRAS1*, *FREM2*, and *GRIP1 *genes, which encode proteins involved in epithelial-mesenchymal adhesion during embryonic development. These proteins play a crucial role in maintaining the integrity of the basement membrane and ensuring proper separation of adjacent epithelial structures. Disruption of this pathway leads to defective morphogenesis, particularly affecting structures that undergo temporary fusion during development. This mechanism explains the characteristic anomalies observed in Fraser syndrome, such as cryptophthalmos or ankyloblepharon (failure of eyelid separation), syndactyly (fusion of digits), and genital malformations. The phenotypic variability of the syndrome may be related to the type and severity of the underlying genetic mutations [10,11].

Clinically, Fraser syndrome is defined by the association of major and minor malformations, and diagnosis relies on established clinical criteria (Table 1). The most frequently reported features include syndactyly and ocular anomalies, particularly cryptophthalmos, described in up to 61.5% of cases, along with genital, renal, and auricular anomalies reported in 59%, 45.3%, and 17.1% of cases, respectively [9,12,13]. Ano-rectal malformations are observed in approximately 29% of patients, while cardiovascular anomalies, although less frequent, are well documented [9,12,13]. Orofacial and neurological manifestations are more variably described [14]. Clinical diagnosis of Fraser syndrome is established if there is the presence of at least two major criteria and one minor criterion, or the presence of one major criterion and at least four minor criteria.

**Table 1:** Diagnostic criteria of the Fraser syndrome. Original table created by the authors based on the diagnostic criteria reported by Thomas et al. [7].

Major criteria	Minor criteria
Cryptophthalmos	Congenital nasal malformations
Syndactyly	Congenital ear malformations
Genital anomaly	Congenital laryngeal malformation
Family history of Fraser syndrome (first degree)	Labial ± palatine cleft
	Urinary and renal tract anomalies
	Musculoskeletal anomalies
	Umbilical hernia
	Learning disabilities

In comparison with these data, our patient presented with several characteristic major features, including bilateral anophthalmia, syndactyly, and genital anomalies, associated with minor criteria such as low-set ears and anal imperforation, thus fulfilling the diagnostic criteria for Fraser syndrome. However, unlike the more frequently reported cryptophthalmos, our case was notable for bilateral anophthalmia, highlighting the spectrum of ocular involvement. Furthermore, the absence of major renal anomalies in our patient contrasts with the relatively high frequency of renal involvement reported in the literature and may partly explain a more favorable immediate outcome.

Prenatal diagnosis of Fraser syndrome, which may be suspected as early as the 13th week of gestation, relies mainly on ultrasound findings such as oligohydramnios and bilateral renal agenesis with increased pulmonary echogenicity [4,15]. However, despite these described features, prenatal recognition remains challenging, and only a limited number of cases have been reported [16]. In our case, no prenatal diagnosis was established, possibly due to the absence of severe renal involvement, further illustrating the variability of prenatal expression and the limitations of antenatal screening.

Several differential diagnoses were considered in view of the polymalformative presentation observed in our patient.

Lenz microphthalmia syndrome, an X-linked disorder, may present with microphthalmia or anophthalmia associated with skeletal and urogenital anomalies. However, it was considered unlikely in our case due to the absence of typical skeletal abnormalities and the presence of multiple major criteria consistent with Fraser syndrome.

Oculo-dento-digital dysplasia is another condition that can involve craniofacial anomalies and limb malformations. Nevertheless, it is usually characterized by dental abnormalities, distinctive facial features, and syndactyly affecting primarily the fingers rather than the toes, which were not observed in our patient.

Ciliopathies, including syndromes such as Meckel-Gruber syndrome or short rib-polydactyly syndromes, were also considered due to overlapping features such as genital and renal anomalies. However, the absence of key features such as polydactyly, severe thoracic hypoplasia, or consistent renal dysplasia, along with the presence of characteristic findings such as ankyloblepharon and syndactyly, made these diagnoses less likely.

Overall, the combination of clinical features observed in our patient was more consistent with Fraser syndrome based on the established diagnostic criteria [7].

Management of Fraser syndrome requires a multidisciplinary approach tailored to the type and severity of malformations. Surgical management, particularly reconstructive and plastic procedures, plays a central role in improving functional outcomes and quality of life. In our patient, planned interventions included correction of syndactyly, genital reconstruction, and management of anal imperforation. In addition, genetic counseling remains essential to inform the family about recurrence risk.

The prognosis of Fraser syndrome is highly variable and largely depends on the extent of visceral involvement. Mortality rates have been reported to reach 25% in stillbirths and 20% within the first year of life, mainly due to severe renal anomalies or airway malformations such as laryngeal atresia [17,18]. In contrast to these severe forms, our case, characterized by the absence of life-threatening renal involvement, may have a more favorable short-term outcome. Nevertheless, long-term prognosis remains uncertain, as surviving patients may experience significant morbidity, including neurodevelopmental impairment.

A limitation of this report is the absence of molecular genetic confirmation, which could have provided additional diagnostic support. However, the diagnosis was considered reliable based on the presence of multiple characteristic clinical features fulfilling established criteria.

Despite this limitation, this case highlights the importance of recognizing atypical presentations of Fraser syndrome, particularly in the absence of renal involvement, and underscores the critical role of detailed clinical evaluation in guiding diagnosis and management in resource-limited settings. 

The clinical features observed in our patient highlight several important aspects of Fraser syndrome that deserve detailed discussion.

The presence of a congenital cardiac anomaly in our patient, specifically an atrioventricular septal defect associated with moderate pulmonary hypertension, represents an important clinical finding. Although cardiac malformations are less frequently reported in Fraser syndrome compared to renal or laryngeal anomalies, they have been described and may significantly impact prognosis. Early identification is essential, as these anomalies may require specialized cardiological follow-up and, in some cases, surgical intervention. In our case, the absence of severe hemodynamic compromise at birth was a favorable element, but close monitoring remains necessary.

Genital anomalies, including ambiguous genitalia, are well-recognized features of Fraser syndrome and may pose significant diagnostic and management challenges. In our patient, the presence of a scrotal-like structure associated with clitoromegaly and labial hypoplasia required careful evaluation. Such findings necessitate a multidisciplinary approach involving pediatric endocrinology, surgery, and genetics, in order to establish an accurate diagnosis, guide sex assignment when necessary, and plan appropriate surgical management. These anomalies also have important psychosocial and ethical implications that must be considered during long-term follow-up.

Notably, our patient did not present with renal agenesis or major renal anomalies, which are among the most severe and life-threatening features of Fraser syndrome. The absence of renal involvement is considered a favorable prognostic factor, as renal agenesis is a major cause of perinatal mortality in this condition. This finding may partly explain the relatively stable early clinical course observed in our patient and suggests a potentially better long-term outcome, although continued surveillance remains essential.

## Conclusions

Fraser syndrome is a rare and complex polymalformative disorder with significant clinical variability. In our case, the diagnosis was strongly suggested by the association of multiple characteristic clinical features, including ocular anomalies, syndactyly, and genital malformations. Notably, the absence of renal involvement in our patient represents an atypical but favorable prognostic feature.

This case highlights the importance of recognizing suggestive clinical findings, which may contribute to an early diagnosis, particularly in resource-limited settings where access to molecular genetic testing is restricted. However, our observations should be interpreted with caution, given the inherent limitations of a single case report.

The absence of molecular genetic confirmation represents an important limitation, as current standards recommend genetic testing for definitive diagnosis when available.

Overall, this report may contribute to improving awareness of the phenotypic spectrum of Fraser syndrome and underscores the importance of a multidisciplinary approach and appropriate genetic counseling in the management of affected patients.
